# Computing H/D-Exchange rates of single residues from data of proteolytic fragments

**DOI:** 10.1186/1471-2105-11-424

**Published:** 2010-08-11

**Authors:** Ernst Althaus, Stefan Canzar, Carsten Ehrler, Mark R Emmett, Andreas Karrenbauer, Alan G Marshall, Anke Meyer-Bäse, Jeremiah D Tipton, Hui-Min Zhang

**Affiliations:** 1 Institut für Informatik, Fachbereich 08, Staudingerweg 9, 55099 Mainz, Germany; 2 Algorithmic Computational Biology, Centrum Wiskunde & Informatica, Amsterdam, The Netherlands; 3 Center for Bioinformatics, Computer Science Department, Saarland University, 66041 Saarbrücken, Germany; 4 Ion Cyclotron Resonance Program, National High Magnetic Field Laboratory, Florida State University, 1800 East Paul Dirac Drive, Tallahassee, FL 32310-4005, USA; 5 Department of Chemistry & Biochemistry, Florida State University, 95 Chieftain Way, Tallahassee, FL 32306, USA; 6 Institute of Mathematics, EPFL, Station 8, 1015 Lausanne, Switzerland; 7 Department of Scientific Computing, Florida State University, Tallahassee FL 32306-4120, USA

## Abstract

**Background:**

Protein conformation and protein/protein interaction can be elucidated by solution-phase Hydrogen/Deuterium exchange (sHDX) coupled to high-resolution mass analysis of the digested protein or protein complex. In sHDX experiments mutant proteins are compared to wild-type proteins or a ligand is added to the protein and compared to the wild-type protein (or mutant). The number of deuteriums incorporated into the polypeptides generated from the protease digest of the protein is related to the solvent accessibility of amide protons within the original protein construct.

**Results:**

In this work, sHDX data was collected on a 14.5 T FT-ICR MS. An algorithm was developed based on combinatorial optimization that predicts deuterium exchange with high spatial resolution based on the sHDX data of overlapping proteolytic fragments. Often the algorithm assigns deuterium exchange with single residue resolution.

**Conclusions:**

With our new method it is possible to automatically determine deuterium exchange with higher spatial resolution than the level of digested fragments.

## Background

In the solution-phase Hyrdogen/Deuterium ex-change (sHDX) experiment, protein surface accessibility is probed by exchange of labile hydrogen for deuterium. Simply speaking, hydrogens located at solvent exposed sites exchange at a higher rate with deuteriums from the solution than others. From these exchange rates one can therefore deduce information about protein solvent accessibility and thus protein conformation.

There is controversy surrounding the effect of *D*_2_*O *solvent on the conformation of proteins. Sheu et al. [1] used molecular dynamic modeling of a small peptide to illustrate compaction of the peptide conformation in *D*_2_*O *versus *H*_2_*O*. This small compaction of the conformation occurs when the pep-tide is fully deuterated (which is never observed in the sHDX experiments). Since sHDX monitors the incorporation of deuterium over time the resulting slight compaction of the structure is minimized. Other methods used for the study of protein/protein interaction or protein conformation such as cross-linking [2,3] or hydroxyl radical addition [4-6] result in large conformational change of the protein structure; leaving sHDX as the method of choice for probing protein conformational changes in solution.

NMR spectroscopy has been the gold standard for determination of protein structure, but it has limitations on protein solubility and molecular weight (<50 *kD*). Solution-phase HDX with mass spectrometry analysis has higher sensitivity and is not limited by molecular weight, but sHDX is hampered with a major difficulty. One only obtains exchange data for peptic fragments and assigning exchange rates to single residues has to be done by manual interpretation.

We provide an automated method to resolve this problem. More precisely, we present an algorithm that enumerates all possible exchange rates for single residues that explain the observed data of the peptic fragments. As the number of possibilities is often very large, we combine sets of assignments to equivalence classes which are easily interpreted such that the number of equivalence classes is typically very small.

The assignment of exchange rates to single residues from the data of the peptic fragments is a combinatorial problem. Hence, we apply methods from combinatorial optimization to it, i.e. we show how to formalize the problem as an integer linear program and propose methods to solve the problem.

## Biochemical Background

Concerning the determination of protein-protein interaction, X-ray crystal diffraction and NMR [7] pro-vide the highest resolution of the sites of interaction. On the downside, both methods require large (milligram) quantities of protein. Other techniques rely on chemical or photo-induced reactions with MS analysis [8,9] to reveal functional groups that are ex-posed to the solvent. These methods also suffer from physical limitations.

Another method utilizes hydroxyl radical reactions with alkyl *CH *bonds. The *OH *tends to re-act mainly with surface-exposed residues providing a good footprint of the solvent exposed surface of the protein(s) [4,6]. The modification is covalent and thus irreversible, but each modification can potentially change the conformation of the protein, thus skewing results.

Exchange of labile hydrogens for deuteriums (sHDX) as a probe of protein surface accessibility does not change the conformation of the protein. Advantages of MS over NMR and X-ray crystallography structural determination are the ability to work at low concentration and high molecular weight.

The experiment is initiated by dilution of the protein solution into a biological buffer made with *D*_2_*O*. Solvent accessible hydrogens are exchanged with deuterium. The exchange is quenched (greatly slowed) by dropping the pH to between pH 2.3 and pH 2.5 and lowering the temperature to approximately 0°C. The protein complex is digested with a protease that is active under quench conditions (such as pepsin) and on-line liquid chromatography is performed directly to the FT-ICR MS. Deuterium in corporation is monitored by the increase in mass of each peptic fragment as the deuterons are added.

**Figure 1:**
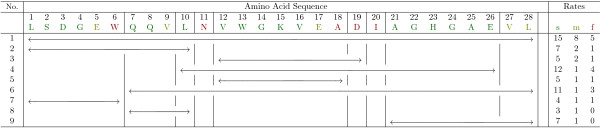
**Sample data set**. Overlap of peptic fragments obtained from our sHDX on myoglobin. The table on the right shows the number of amide hydrogens predicted to be either slow, medium or fast (based on MEM). The vertical lines show the decomposition of the sequence into parts induced by the fragments. We want to automatically draw conclusions on the exchange rates of single amino acids, like the one that the second D residue has to have medium exchange rate, concluded from the restrictions imposed by fragments 3 and 5. Notice that we already deleted the N-terminal amino acid as we cannot see them exchange.

These data sets are large, often with many over-lapping proteolytic fragments. From these data, the exchange rate is easily determined for the same peptic fragments from the protein and the protein/protein complex [10] (all other fragments are disregarded). When peptic fragments are not directly comparable, but are overlapping (Figure 1) manual interpretation must be performed to assign exchange rate to single residues. Data analysis is the greatest bottleneck in sHDX experiments; thus automated data analysis is necessary. Furthermore, we are interested in all such assignments, as averaging over all solutions gives better results in practice.

## Mathematical Abstraction

In this section, we present our mathematical model for the assignment of exchange rates to residues. A brief overview of the introduced terms and symbols can be found in Table 1. In an idealized setting, we consider the following problem. We sequentially number the *n *residues of a protein from1 to *n*, beginning at the N-terminal residue and ending at the C-terminal residue. The set of peptic fragments resulting from the digestion of the protein is captured by a set ℱ of integer intervals (*i*, *j*) := {*k *∈ ℕ | *i *≤ *k *≤ *j*}, for two positive integers *i*, *j *with *i *≤ *j*, representing the endpoints of the corresponding fragment. In other words, the peptic fragment represented by (*i*, *j*) spans residues *i*, *i*+1, ..., *j*. Furthermore, *K *denotes the number of different classes of exchange rates, arising from the discretization of the experimentally measured deuterium uptake rates [11]. The *K *distinct classes of exchange rates, to which we simply refer as colors in the following, are represented by set S. To simplify notation we number the colors from 1 to *K *and identify in the following the colors by their respective number. The experimentally found bulk information of how many residues within each fragment (*i*, *j*) ∈ ℱ fall into each of the exchange rate categories is given by "requirement" integers $b_{\left(i,j\right)}^{k}$, for each fragment (*i*, *j*) ∈ ℱ and each color *k *∈ S. We call the vector **b**^*k *^of "requirements" with respect to color *k*, indexed by fragments from, ℱ the *right hand side *for color *k*. In our experimental data, exactly three different colors are distinguished (interpreted as slow, medium, and fast exchange rates), i.e. *K *= 3. However, our method is not restricted to this case.

**Table 1:** Overview of terms and symbols

Term/Symbol	Meaning
fragment	A fragment is a set of consecutive residues resulting from the digestion of the protein.
ℱ	The set of all (possibly overlapping) fragments.

color	We divide the exchange rates into classes and associate a color with each class.
S	The set of the *K *distinct colors.

part	A part is a maximal set of residues contained in the same set of fragments, i.e., an inclusion-wise maximal subset of a fragment that is either contained in or disjoint from any other fragment.
P	The partition of the residues into parts as defined above.

subproblem	An instance decomposes into independent subproblems if there is no overlap between the fragments of the different subproblems.

Description of the terms and symbols used in the mathematical description.

The mathematical notion introduced above is illustrated in Figure 1. There the residues, numbered from 1 to 28, are spanned by 9 peptic fragments, i.e. |ℱ = 9|. The third peptic fragment "VWGKVEAD" will then be represented by the integer interval (12; 19). From the experimental data we know that 5 out of the 8 residues contained in this fragment exchanged slowly (s), two at medium rate (m), and the last remaining residue exchanged fast (f) and thus $b_{\left(12,19\right)}^{s}=5,\;b_{\left(12,19\right)}^{m}=2$, and $b_{\left(12,19\right)}^{f}=1$.

Determining the exchange rate of single residues from the experimentally found data for the peptic fragments then translates into finding a "consistent" assignment of colors from S to the integer points from {1, ..., *n*}, representing the residues of the protein, that complies with the constraints imposed by the "requirements" $b_{\left(i,j\right)}^{k}$. More precisely, we have to determine an assignment *π *: {1, ..., *n*} ↦ S such that |{*i *≤ *l *≤ *j *: *π*(*l*) = *k*}| = $b_{\left(i,j\right)}^{k}$ for all given fragments (*i*, *j*) ∈ ℱ and all possible colors *k *∈ S. We call such an assignment *feasible*.

We say that two fragments (*i*, *j*) and (*i'*, *j'*) over-lap, if they share at least one common residue, i.e. (*i*, *j*) ∩ (*i'*, *j'*) ≠ ∅. The partition of the set of fragments ℱ into a maximum number of subsets, such that no two fragments from different subsets overlap, defines independent subproblems; an assignment of exchange rates to the residues spanned by the fragments of one subset does not affect the solution of a subproblem corresponding to any other subset of fragments.

Furthermore, we denote by P the partition of the set of residues {1, ..., *n*} into maximal subsets such that residues from the same subset are spanned by exactly the same set of fragments. More precisely, for all residues *i *and *j *in the same part of P and for all fragments *f *∈ ℱ it holds *i *∈ *f *⇔ *j *∈ *f*. Hence, for each part *p *∈ P and each fragment *f *∈ ℱ either *p *⊆ *f *or *p *∩ *f *= ∅. In Figure 1 for example, residues number 7 (Q), 8 (Q), and 9 (V), are all contained in fragments number 1, 2, 6, and 8 and thus form an element $\overset{ª}{p}$ of partition P. Note that for the two neighboring residues the set of containing fragments differs from {1, 2, 6, 8} and therefore part$\overset{ª}{p}$ = {7, 8, 9} is maximal.

However, data collected in real experiments usually contain some noise, such that no feasible assignment of exchange rates as defined above exists. Therefore, the goal is to compute all assignments that minimize the total sum of errors. Here, the error of an assignment *π *in fragment (*i*, *j*) ∈ ℱ with respect to color *k *is defined as the surplus, respectively the shortage, of residues in (*i*, *j*) that are assigned color *k*, compared to the number of such residues suggested by the experimental data. That is,

(1) $$e_{\left(i,j\right)}^{k}=|b_{\left(i,j\right)}^{k}-|\{i\leq l\leq j:\pi (l)=k\}||$$

and thus the objective is to minimize the sum of this deviations over all fragments and colors, i.e.

(2) $$\text{minimize}\sum_{k\in S}\sum_{\left(i,j\right)\in ℱ}e_{\left(i,j\right)}^{k}.$$

In Figure 1 the colors green, yellow and red encode an optimal assignment π* of the exchange rates slow, medium and fast, with respect to objective (2). Under this assignment, fragment 3 contributes an error of 1 both w.r.t. color yellow (medium exchange rate)and color red (fast rate) to the total error of 17, while it satisfies the requirement for color green (slow rate, numbered 1) $b_{\left(12,19\right)}^{1}=5$ exactly.

## Results and Discussion

In the following, we present different approaches to tackle the assingment problem that we have derrived from the mathematical abstraction mentioned before.

### Integer linear programming formulation

First, we formulate the idealized version of the problem assuming error-free experimental data as an integer linear program (ILP). That is, we give an ILP whose feasible solutions correspond one-to-one to the feasible assignments of colors to residues.

Let *π *: {1, ..., *n*} ↦ S be an assignment of colors to residues. A binary variable $x_{i}^{k}$ for every color *k *∈S and every residue *i *∈ {1, ..., *n*} indicates whether residue *i *is assigned color *k *or not, i.e.

$$x_{i}^{k}=\{\begin{array}{l} 1\text{if}\;\pi (i)=k\\ 0\text{otherwise}. \end{array}$$

We denote by $x^{k}=\left(x_{1}^{k},x_{2}^{k},...,x_{n}^{k}\right)$ the vector of binary variables modeling the assignment of color *k *and let $x={\left(x^{k}\right)}_{k\in S},\;\;x\in {\left\{0,1\right\}}^{Kn}$.

Since every residue is assigned exactly one color, it must hold $\sum_{k\in S}x_{i}^{k}=1$ for all *i *∈ {1, ..., *n*}. Conversely, every 0-1 assignment to variables **x **satisfying $\sum_{k\in S}x_{i}^{k}=1$ for all *i *∈ {1, ..., *n*} corresponds to an assignment of colors to residues. A 0-1 assignment to **x **corresponds to a feasible color assignment *π*, if and only if furthermore $\sum_{l=i}^{j}x_{l}^{k}=b_{\left(i,j\right)}^{k}$ holds for all (*i*, *j*) ∈ ℱ and *k *∈ S.

Now consider the problem of computing an assignment with minimum total error. Translating the definition of the error that we make when assigning color *k *(or not) to residues in fragment (*i*, *j*) (see equation (1)) to the context of 0-1 assignments to variables **x**^*k*^, the problem of minimizing (2) becomes

$$\text{minimize}\sum_{k\in S}\sum_{\left(i,j\right)\in ℱ}|b_{\left(i,j\right)}^{k}-\sum_{l=i}^{j}x_{l}^{k}|.$$

Concerning the formulation of a minimum sum of absolute values in terms of a linear objective function and linear constraints, observe that $\left|b_{\left(i,j\right)}^{k}-\sum_{l=i}^{j}x_{l}^{k}\right|$ is the smallest number $e_{\left(i,j\right)}^{k}$ that satisfies

$$\begin{array}{c} e_{\left(i,j\right)}^{k}\geq \sum_{l=i}^{j}x_{l}^{k}-b_{\left(i,j\right)}^{k}\;\text{and}\\ e_{\left(i,j\right)}^{k}\geq -\sum_{l=i}^{j}x_{l}^{k}+b_{\left(i,j\right)}^{k} \end{array}$$

Hence, after introducing a variable $e_{\left(i,j\right)}^{k}$ for every color *k *∈ S and every fragment (*i*, *j*) ∈ ℱ, the integer linear program we are looking at is

$$\begin{array}{lll} \min & \sum_{k\in S}\sum_{(i,j)\in ℱ}e_{\left(i,j\right)}^{k} & \\ \text{s}.\text{t}. & e_{\left(i,j\right)}^{k}\geq \sum_{l=i}^{j}x_{l}^{k}-b_{\left(i,j\right)}^{k} & \text{for}\;\text{all}\;k\in S,(i,J)\in ℱ\\ & e_{\left(i,j\right)}^{k}\geq -\sum_{l=i}^{j}x_{l}^{k}+b_{\left(i,j\right)}^{k} & \text{for}\;\text{all}\;k\in S,(i,J)\in ℱ\\ & \sum_{k\in S}x_{l}^{k}=1 & \text{for}\;\text{all}\;1\leq l\leq n\\ & x\in {\left\{0,1\right\}}^{Kn} & \end{array}$$

We refer to this integer linear program as *basic-ILP*.

In our experiments, it turns out that finding a single solution is very fast, whereas enumerating all solutions takes quite some time due to their large number. This large number can be explained as follows: Recall that P is the partition of {1, ..., *n*} into a minimal number of parts, such that for each element *p *∈ P and each fragment *f *∈ *F *either *p *⊆ *f *or *p *∩ *f *= ∅. In other words, no fragment starts or ends within such a part. Therefore, from an assignment *π *we can derive further assignments *π' *exhibiting the same total error, by simply permuting the colors within these parts, i.e. if *i*, *j *∈ *p *for *p *∈ P and the total error of an assignment *π *is *e*_1_, than *π' *with *π' *(*i*) = *π *(*j*), *π' *(*j*) = *π *(*i*) and *π' *(*l*) = *π *(*l*) for *l *≠ *i, j *has total error *e*_2 _with *e*_2 _= *e*_1_. We call two assignments *equivalent*, if one can be obtained from the other by iteratively applying this rule.

In order to enumerate equivalent solutions only once, we modify our integer linear program as follows: For *k *∈ S and *p *∈ P, we replace the binary variables ${\left(x_{l}^{k}\right)}_{l\in p}$ by a single integer variable $y_{p}^{k}$ with $y_{p}^{k}:=\sum_{l\in p}x_{l}^{k}$. Moreover, let *A *be the |ℱ| × |P| inclusion matrix, i.e. for every *f *∈ ℱ and *p *∈ P, the corresponding entry is given by

$$a_{f,p}=\{\begin{array}{cc} 1 & \text{if }p\subseteq f\\ 0 & \text{otherwise}. \end{array}$$

We denote by $e^{k}={\left(e_{\left(i,j\right)}^{k}\right)}_{(i,j)\in ℱ}$ the vector of errors with respect to color *k *and by $y^{k}={\left(y_{p}^{k}\right)}_{p\in }$ the number of residues colored *k*. In matrix notation the constraints are then of the form

$$\begin{array}{c} -Ay^{k}+e^{k}\geq -b^{k}\\ Ay^{k}+e^{k}\geq b^{k} \end{array}$$

for all *k *∈ S. Hence our integer linear program be-comes

(3) $$\begin{array}{lll} \min & \sum_{k\in S}\sum_{f\in ℱ}e_{f}^{k} & \\ \text{s}.\text{t}\text{.} & -Ay^{k}+e^{k}\geq -b^{k} & \text{for}\;\text{all}\;k\in S\\ & Ay^{k}+e^{k}\geq b^{k} & \text{for}\;\text{all}\;k\in S\\ & \sum_{k\in S}y^{k}=P & \\ & y\geq 0,\;\text{integer} & \end{array}$$

where **P **is the vector that contains |*p*| for each component *p *∈ Pand $y={\left(y^{k}\right)}_{k\in S}$ We refer to this integer linear program as *improved-ILP*. We compute all solutions within a certain error bound by following basically the same approach as described above. However, the number of solutions now is just a fraction of the number of solutions of the original *basic-ILP *yielding a significant speed-up

Although there is commercial software for integer programming which quickly solves instances of reasonable size, there is no algorithm that is guaranteed to find an optimum solution in polynomial time, since integer programming is NP-complete in general. However, the problem of assigning exchange rates to residues in a way that is conform with the experimentally found bulk data exhibits a certain combinatorial structure. In the next section, we exploit this fact to derive an exact polynomial-time algorithm for the case of two colors and use it as a building block for approximation algorithms for more than two colors subsequently.

### A Combinatorial Approach

First, let us consider the special case of two colors, i.e. *K *= 2 and thus S = {1,2}. That is, we have constraints of the form $y_{p}^{1}+y_{p}^{2}=\left|p\right|$ for all *p *∈ P. This allows us to simplify the linear program considerably. We replace $y_{p}^{2}=\left|p\right|-y_{p}^{1}$ and omit the superscript of the y-variables in the following. This yields

$$\begin{array}{cc} -Ay+e^{1}\geq -b^{1} & Ay+e^{2}\geq F-b^{2}\\ Ay+e^{1}\geq b^{1} & \;-Ay+e^{2}\geq -F+b^{2} \end{array}$$

where **F **is the vector of fragment sizes. We may get rid of half of the constraints by the following observation. Let **b **:= max {**b**^1^, **F **- **b**^2^} and $\overset{ª}{b}:=\min\{b^{1},F-b^{2}\}$ where the maximum is taken component-wise. Let **y **be an arbitrary feasible solution with minimum total error $\sum_{f\in ℱ}e_{f}^{1}+e_{f}^{2}$. We may consider the contribution of each fragment independently for that particular **y**. We may rename the error variables **e**^1 ^and **e**^2 ^component-wise according to **b **and $\overset{ª}{b}$, i.e.

(4) $$\begin{array}{cc} e_{f}:=\{\begin{array}{ll} e_{f}^{1} & \text{if}\;b_{f}=b_{f}^{1}\\ e_{f}^{2} & \text{otherwise} \end{array} & {\bar{e}}_{f}:=\{\begin{array}{ll} e_{f}^{1} & \text{if}\;{\bar{b}}_{f}=b_{f}^{1}\\ e_{f}^{2} & \text{otherwise} \end{array} \end{array}$$

For each *f *∈ ℱ with ${\overset{ª}{b}}_{f}\leq a_{f}^{T}y\leq b_{f}$, we have $e_{f}^{1}+e_{f}^{2}=b_{f}-{\overset{ª}{b}}_{f}$. If $a_{f}^{T}y>b_{f}$, we get $e_{f}^{1}+e_{f}^{2}=2e_{f}+b_{f}-{\overset{ª}{b}}_{f}$. Analogously, we get $e_{f}^{1}+e_{f}^{2}=2{\overset{ª}{e}}_{f}+b_{f}-{\overset{ª}{b}}_{f}$ if $a_{f}^{T}y<{\overset{ª}{b}}_{f}$. Hence, it is sufficient to optimize the following linear program

(5) $$\begin{array}{ll} \min & \sum_{f\in ℱ}e_{f}+{\bar{e}}_{f}\\ \text{s}.\text{t}. & -Ay+e\geq -b\\ & Ay+\bar{e}\geq \bar{b}\\ & -y\geq -P\\ & y,e,\bar{e}\geq 0 \end{array}$$

which is integral if **b **and $\overset{ª}{b}$ are integral since the constraint matrix is totally unimodular. The corresponding dual LP is given by

(6) $$\begin{array}{ll} \max & -b^{T}f^{1}-{\bar{b}}^{T}f^{2}+P^{T}f^{3}\\ \text{s}.\text{t}. & -A^{T}f^{1}+A^{T}f^{2}-f^{3}\leq 0\\ & 0\leq f^{1,2}\leq 1\\ & 0\leq f^{3} \end{array}$$

which is equivalent to (multiplying the objective function by -1 and introducing slack variables)

(7) $$\begin{array}{ll} -\min & -b^{T}f^{1}-{\bar{b}}^{T}f^{2}+P^{T}f^{3}\\ \text{s}.\text{t}. & -A^{T}f^{1}+A^{T}f^{2}-f^{3}+f^{4}=0\\ & 0\leq f^{1,2}\leq 1\\ & 0\leq f^{3,4} \end{array}$$

We will show next that this LP is a Minimum Cost Circulation Problem. To this end, let *M *be the matrix of the equality constraints, i.e.

$$M\;:=\;(-A^{T}\;A^{T}\;-I\;I).$$

**Figure 2:**
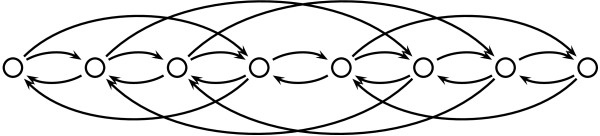
**Fragment graph example**. Example of a fragment graph with |P| = 7 The corresponding fragments are (1, 3), (2, 5), (3, 6), and (5, 7).

Note that this matrix has the column-wise consecutive-ones property. By row operations like in Gaussian elimination, we can easily transform *M *such that each column contains exactly one +1 and one -1, as follows. We add the dummy constraint 0 = 0 at the end and subtract from each row its predecessor. The resulting matrix, say $\overset{ª}{M}$, can be considered as the node-arc-incidence matrix of a directed graph. Since the right hand side remains unchanged, we get a Minimum Cost Circulation problem on a graph with |P| + 1 nodes and O(||+|ℱ|) arcs [12]. As a matter of fact, we have for each variable *y_p _*two arcs corresponding to the constraint 0 ≤ *y_p _*≤ |*p*| and for each fragment (*i*, *j*) the arcs (*i*, *j *+ 1) and (*j *+ 1, *i*) as depicted in Figure 2.

For three or more colors the complexity is open. The totally unimodularity of the constraint matrix is destroyed, i.e. there are instances with fractional vertices, e.g. the one from Figure 2 with the appropriate right hand sides. Moreover, there is an instance which has a positive error, but the value of the LP is 0. Hence the integrality gap is infinite. If the number of colors is not fixed but part of the input, the problem is NP-complete [13].

### A Simple and Efficient Heuristic for the General Case

We present an algorithm that uses our combinatorial approach for the 2-color case (*K *= 2) from previous section as a subroutine to provide solutions that approximate (without performance guarantee) a coloring, i.e. an assignment of colors to residues, with minimum total error for instances with arbitrary but fixed number of colors. The general idea is to reduce the problem to the 2-color case by merging all but one color, say color *i*, to a single color and solve the resulting problem by an algorithm for the minimum cost circulation problem, as described in the section about the Combinatorial Approach. We remove residues colored *i *by the obtained solution and solve the coloring problem on the remaining residues using *K *- 1 colors recursively.

Our approach works as follows. Consider an arbitrary color *k *∈ S. We compute a subset of the residues that are assigned color *k *such that the total error with respect to color *k *and the sum of all remaining colors is minimized, i.e. we solve the two color problem with requirements (= right hand sides)

$$(b^{k},\sum_{k^{\prime }\neq k}b^{k^{\prime }}).$$

Residues assigned color *k *in an optimal solution to this problem will be colored *k *in the final solution too, the assignment of the remaining colors S\{*k*} to the remaining residues is computed recursively.

Note that the order in which colors are selected to be the next fixed color *k *in the recursive computation can be arbitrary. Nevertheless, they might lead to solutions of different total error. As we have only three different colors in our experimental data, we evaluate all six orderings and return the best solution found.

In the next section we present a Lagrangian relaxation method to compute, based on our combinatorial approach for the 2-color case, a bound on the minimum total error, which is exploited in a branch-&-bound manner to determine all optimal colorings.

### A Lagrangian Relaxation Approach

In this section we propose a *Lagrangian relaxation *approach for the problem, which is particularly suit-able for finding *all *optimal solutions. It is based on the *improved-ILP *formulation:

(8) $$\min\sum_{k\in S}\sum_{f\in ℱ}e_{f}^{k}$$

(9) $$\begin{array}{ccc} \text{s}.\text{t}. & -Ay^{k}+e^{k}\geq -b^{k} & \text{for}\;\text{all}\;k\in S \end{array}$$

(10) $$\begin{array}{ll} Ay^{k}+e^{k}\geq b^{k} & \text{for}\;\text{all}\;k\in S \end{array}$$

(11) $$\begin{array}{l} \sum_{k\in S}y^{k}=P\\ y\geq 0,\;\text{integer} \end{array}$$

where **P **is the vector that contains the length of parts in P. The problem can be considered to contain independent structures for each color *k *∈ S, namely the set of positive integer vectors **y**^*k *^satisfying (9) and (10) under the objective (8), that are linked by constraints (11). Therefore, dualizing the linking constraints (11), with Lagrangian multipliers *λ*, splits the problem into an independent problem for each color *k *∈ S:

$$\begin{array}{lll} \min & \sum_{k\in S}\sum_{f\in ℱ}e_{f}^{k}+\lambda^{T}\left(\sum_{k\in S}y^{k}-P\right) & \left(IP(\lambda )\right)\\ \text{s}.\text{t}. & -Ay^{k}+e^{k}\geq -b^{k} & \text{for}\;\text{all}\;k\in S\\ & Ay^{k}+e^{k}\geq b^{k} & \text{for}\;\text{all}\;k\in S\\ & 0\leq y^{k}\leq P & \text{for}\;\text{all}\;k\in S\\ & y\;\text{interger} & \end{array}$$

Neglecting the constant term -*λ*^*T*^**P **in the objective function and replacing error variable *e *by *e *+ *ē *we have to determine, for every color *k *∈ S, an optimal integral solution to the following linear program:

(12) $$\begin{array}{l} \min\;\;\;\sum_{f\in ℱ}\left(e_{f}^{k}+{\overset{ª}{e}}_{f}^{k}\right)+\lambda^{T}y^{k}\\ \text{s}.\text{t}.\;\;\;\;-Ay^{k}+e^{k}\geq -b^{k} \end{array}$$

(13) $$Ay^{k}+{\overset{ª}{e}}^{k}\geq b^{k}$$

(14) $$\begin{array}{l} e^{k},{\overset{ª}{e}}^{k}\geq 0\\ 0\leq y^{k}\leq P \end{array}$$

Note that we added constraint (14) to enforce $e_{f}^{k}$ or ${\overset{ª}{e}}_{f}^{k}$ to be zero if ${\overset{ª}{e}}_{f}^{k}$, respectively $e_{f}^{k}$, corresponds to the absolute value of the error, i.e. if the constraint (13), respectively the constraint (12), for fragment *f *is tight. Note that we have to enforce $e_{f}^{k}$ and ${\overset{ª}{e}}_{f}^{k}$ to be nonnegative. In every optimum solution either **e **or **ē **(or both) will be zero for each fragment f. Similar as for linear program (5), its dual is given by (omitting the color superscript *k*):

(15) $$\begin{array}{ll} -\min & b^{T}f^{1}-b^{T}f^{2}+P^{T}f^{3}\\ \text{s}\text{.t}\text{.} & -A^{T}f^{1}+A^{T}f^{2}-f^{3}+f^{4}=\lambda \\ & 0\leq f^{1,2}\leq 1\\ & 0\leq f^{3,4} \end{array}$$

This linear program differs from LP (7) only in the right-hand sides of the equality constraints.

## Conclusions

We applied our methods to process data from typical biochemical experiments. We report our results for four proteins: Calcium-binding protein (Cabin), Cytochrome P450 (CytoC), FK506 binding protein(FKBP), with two different digests (pepsin and XIII), and myoglobin. As a preprocessing step, the single fragments were analyzed with our integer linear programming based technique [14], except for FKBP V2 (MEM) which was analyzed with the MEM-method [15] and is based on the same data as FKBP V1 (ILP). We analyzed FKBP with only the xiii digestion (V3) and combined the datasets from the two digestions (V1, V2 and V4). The number discretized exchange rates per fragment obtained in this preprocessing step serves as input to the algorithm.

The instances have between 74 and 152 residues and between 18 and 49 fragments. The solutions with a minimal number of errors could be computed in less than 0.11 seconds for all instances. Computing all (non-equivalent) solutions with a minimal number of errors, from 96 up to almost 20 million in number, took less than 7 minutes, where the running time greatly depends on the number of solutions (see Table 2). Computing all solutions using the basic-ILP takes much longer as with the improved-ILP. For all instances, the heuristic computes a solution with the minimal error.

**Table 2:** Results & Runtime

	Instance						Improved-ILP		Lagrange		Heuristic
**Name**	***n***	P	***n*/**P	ℱ	**ϵ**	***T***_**1**_	***T***_***all***_	***#*-Sol**	***T***_**1**_	***T***_***all***_	***T***_**1**_

Cabin	78	26	3.0	34	128	0.02	3.25	36	1.36	8.35	0.02

CytoC	74	18	4.1	17	40	0.03	0.37	1980	0.27	6.10	0.01

Subproblem 1	27	5	5.4	6	6	0.01	0.01	1	0.01	0.01	0.003
Subproblem 2	26	5	5.2	6	30	0.01	0.32	110	0.17	5.81	0.004
Subproblem 3	15	6	2.5	5	4	0.01	0.04	18	0.09	0.28	0.004

FKBP V1 (ilp)	101	34	3.0	31	47	0.04	1.18	37800	1.03	137.36	0.017

Subproblem 1	35	15	2.3	12	15	0.01	0.45	126	0.57	32.83	0.009
Subproblem 2	16	5	3.2	5	4	0.01	0.02	4	0.04	0.05	0.003
Subproblem 3	36	12	3.0	14	28	0.02	0.71	75	0.42	104.48	0.005

FKBP V2 (mem)	101	34	3.0	31	46	0.03	13.82	1160040	2.03	560.56	0.02

Subproblem 1	35	15	2.3	12	16	0.01	4.41	840	1.26	305.4	0.007
Subproblem 2	16	5	3.2	5	2	0.01	0.01	1	0.01	0.01	0.002
Subproblem 3	36	12	3.0	14	28	0.01	9.4	1381	0.76	255.15	0.007

FKBP V3 (xiii)	103	34	3.0	47	38	0.05	0.16	6	0.14	0.13	0.026

Subproblem 1	22	10	2.2	16	12	0.01	0.04	1	0.02	0.03	0.008
Subproblem 2	10	4	2.5	4	2	0.01	0.02	3	0.02	0.02	0.002
Subproblem 3	11	5	2.2	4	0	0.01	0.01	1	0.02	0.01	0.003
Subproblem 4	25	10	2.5	22	24	0.01	0.08	2	0.07	0.06	0.008
Subproblem 5	3	1	3.0	1	0	0.01	0.01	1	0.01	0.01	0.001

FKBP V4 (both)	105	43	2.4	56	58	0.05	0.96	1536	0.88	7.15	0.032

Subproblem 1	49	20	2.5	24	18	0.02	0.55	24	0.8	6.08	0.012
Subproblem 2	11	5	2.2	4	0	0.01	0.01	2	0.02	0.01	0.003
Subproblem 3	25	12	2.1	26	40	0.01	0.39	16	0.5	1.05	0.009
Subproblem 4	4	3	1.3	2	0	0.01	0.01	2	0.01	0.01	0.002

Myoglobin	152	49	3.1	48	42	0.1	0.98	1121760	1.13	13.25	0.023

Subproblem 1	17	9	1.9	10	14	0.02	0.16	20	0.22	1.98	0.004
Subproblem 2	12	2	6.0	4	2	0.01	0.01	2	0.01	0.01	0.002
Subproblem 3	22	8	2.8	8	8	0.01	0.26	82	0.34	8.56	0.005
Subproblem 4	37	14	2.6	17	14	0.01	0.49	38	0.45	2.51	0.009
Subproblem 5	3	1	3.0	1	0	0.01	0.01	1	0.01	0.01	0.002
Subproblem 6	21	6	3.5	6	4	0.02	0.03	9	0.08	0.16	0.003
Subproblem 7	4	1	4.0	1	0	0.01	0.01	1	0.01	0.01	0.001
Subproblem 8	7	1	7.0	1	0	0.01	0.01	1	0.01	0.01	0.002

We give the characteristics of the instance, i.e., the number of residues (*n*), the number of fragments (|ℱ|), the number of non-equivalent parts (|P|), the average length of these parts (*n*/|P|) and the minimal error of an solution (ϵ). We give the solution times in seconds to compute one (*T*_1_) and all (*T_all_*) solutions and the number of solutions found (#-Sol) all with respect to the improved-ILP. Furthermore we give the running times for our Lagrangian approach and the error and running time of the heuristic.

**Figure 3:**
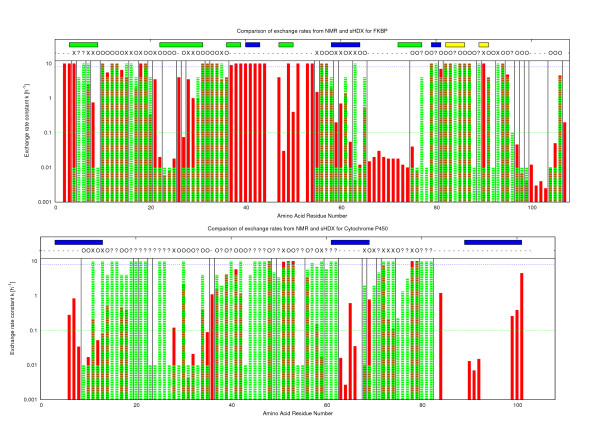
**Single residue results for FKBP and cytochrome C**. Results for FKBP (top) and cytochrome C (bottom) at single residue resolution. Rates from NMR are colored in red, our results from sHDX are colored in green. Vertical Black lines denote the boundary between consecutive parts. Legend for Symbols: '0' Rates agree on position, 'X' Rates disagree,'?' NMR data is missing, '-' sHDX data is missing. The horizontal lines indicate the range of the discretized values. slow ≤ 0.1 *h*^-1^, and fast ≥ 8 *h*^-1^. Secondary structure is indicated by the horizontal bars on the top: alpha helical in blue, beta sheets in green and loops in yellow.

Where available, we compared our assignments of exchange rates to the results obtained by NMR-analysis (FKBP and CytoC [16]). The error measure is based on a comparison per part. Within each part, the rates assigned by the algorithm are compared to the ones from NMR. Table 3 summarizes the results. The table also shows the importance of taking all solutions into account, as averaging typically yields better results than a single solution. The assignments coincide to 60 - 75% to the ones obtained by NMR, when choosing the optimal ordering with in the parts of equivalent residues. Figure 3 provides the results for FK506 and Cytochrome P450 at single residue resolution for manual inspection.

**Table 3:** Comparison with NMR

Dataset	Single solution	Majority Vote	Arithmetic Mean
CytoC	77.87	69.45	69.45
FKBP V1 (ilp)	58.03	67.09	74.69
FKBP V2 (mem)	67.09	67.68	67.09
FKBP V3 (xiii)	75	71.88	70.32
FKBP V4 (both)	58.03	62.97	64.20

Comparison of our results to those obtained by NMR. The error measure is based on an comparison per part, hence taking the sequence positions into account. To obtain an unique answer, we used two methods to average over all solutions, namely taking the majority and the average. Then we counted the percentage of amino acids that have the same exchange rates in both methods, according to the optimal reordering within the parts of equivalent residues.

**Figure 4:**
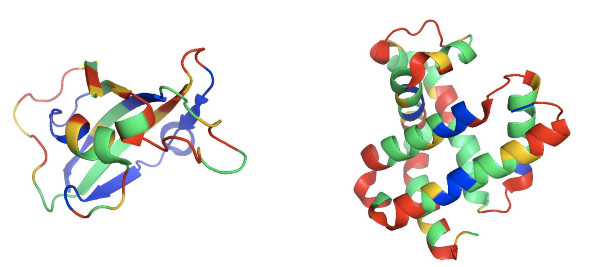
**Structural view of results**. The exchange rates (fast, medium slow) are color-coded (red, yellow, green) in the structural view. The color blue means that we have no fragments covering this part and hence we do not predict anything. We present FKBP on the left and Myoglobin on the right.

A structural view on the results for FK506 and myoglobin is given in Figure 4. For myoglobin we do not have NMR data at hand. Nevertheless does the figure nicely agree with the expected out come, as buried parts of the protein show on the average lower exchange rates than exposed parts. The two figures have been produced by use of PyMOL [17].

In our solutions, the resolution is significantly increased compared to the input data, i.e., the length of fragments obtained from the sHDX experiments. The parts are typically small (see Table 2), between2 and 4 residues. 75% of the parts are smaller than 8 residues. For 46% of the amino-acids, we get single-residue resolution on the data.

The results for the real instances are very promising, as the small number of easily interpretable classes of equivalent solutions can be used in protein structure prediction tools and for manual inspection.

## Methods

In this Section, we describe the computational methods, which we use to solve the different formulation, as well as the biochemical methods to obtain the experimental data.

### Solution of the integer linear program

We implemented our approach using the C++-Library SCIL [18] to solve integer linear programs. SCIL uses the libraries LEDA [19] and SCIP [20].SCIP uses CPLEX [21] or SoPlex [22] as solver for linear programs. The underlying solution method is branch-&-bound, that is described in detail in [23].

In order to find all solutions within a given error bound *e*, the constraint

$$\sum_{k\in S}\sum_{(i,j)\in ℱ}e_{\left(i,j\right)}^{k}\leq e$$

is added to the integer linear program and hence we are faced with the problem of computing all feasible solutions of an integer linear program. We do this with a branching-approach similar to the classical branch-&-bound method for finding an optimal solution: First, the linear relaxation is solved. If the linear relaxation is infeasible, the search on this branch terminates. If the solution is integral, it is stored (provided the solution was not found yet). If there is a binary variable which was not fixed so far (i.e. not set to 0 or 1), one such variable $x_{l}^{k}$ is picked and the two subproblems, in which the variable is fixed o 0 or 1 recursively, are solved. Notice that it is possible that we branch on a variable which already has an integral value. In this case, the solution of the linear relaxation of the subproblem will be the same as in problem itself. Nevertheless, we will terminate, as there are only a finite number of variables to branch on.

### Solving the Combinatorial Problem

We may use any algorithm that solves the Minimum Cost Circulation problem, e.g. Cycle Canceling or Successive Shortest Path (see [12] for further reference). Both approaches have their advantages. The former always maintains a feasible circulation, i.e. we start with the zero flow and augment flow along negative cycles in the residual network until no negative cycle remains. Since the residual network with respect to an optimal circulation does not contain a directed negative circuit, we can find node potentials, i.e. a corresponding dual solution, using the Bellman-Ford algorithm in O(|P| ⋅ |ℱ|) time. The difference between the potential of two neighboring nodes then yields the value of the corresponding *y*-variable. The errors are determined straight forward. If there is a solution without error this approach yields a solution within the running time of Bellman-Ford. On the other hand, the Successive Shortest Path algorithm maintains similar node potentials such that the arc-weights remain non-negative. Since the total excess is bounded by |P| in our case, the running time of that algorithm is $O\left(\left|P|\cdot |ℱ|+|P{|}^{2}\log|P\right|\right)$.

### Solving the Lagrangian Dual

Instead of *a minimum cost circulation *problem(right-hand side is **0**), we have to solve the more general *minimum cost flow *problem [12] where the supplies and demands $\overset{ª}{\lambda }$ of the nodes are determined by the difference of Lagrangian multipliers, i.e. $\overset{ª}{\lambda }$ is of dimension |P| + 1 and ${\overset{ª}{\lambda }}_{i}=\lambda_{i}-\lambda_{i-1}$ for $2\leq i\leq \;|P|,{\overset{ª}{\lambda }}_{1}=\lambda_{1}$ and ${\overset{ª}{\lambda }}_{|P|+1}=-\lambda_{\left|P\right|}$. A feasible flow of minimum cost can be computed efficiently by, e.g., the *cycle-canceling algorithm *and the *successive shortest path algorithm*, as well as variants of them, like the *capacity scaling algorithm *[12]. In our implementation (C++) we used the LEDA library [19] to solve the Lagrangian subproblem by analgorithm based on capacity scaling and successive shortest path computation [12].

We improve the resulting bounds by the subgradient optimization method described in the following and incorporate the overall approach into a branch-&-bound algorithm as the lower bounding scheme.

Let *v*(*IP *(*λ*)) denote the optimal value of *IP *(*λ*). Then for any vector *λ *of Lagrangian multipliers, the (non-differentiable) Lagrangian function

z(λ)=v(IP(λ))

provides a lower bound on the minimum total error. To benefit from the sharpest possible bound in the branch-&-bound framework we are interested in solving the Lagrangian dual problem

$$z^{*}=\underset{\lambda }{\max}z(\lambda ).$$

We apply the subgradient method to obtain near-optimal Lagrangian multipliers. Following the approach by Held and Karp [24] we iteratively determine values *λ*^*ℓ*+1 ^for *ℓ *= 0,1, ..., of the Lagrangian multipliers by moving in the direction of a subgradient with "step length" *μ_ℓ_*:

$$\lambda^{\ell +1}=\lambda^{\ell }+\mu_{\ell }\left(\sum_{k\in S}y^{k}(\ell )-P\right),$$

where ${\left(y^{k}(\ell )\right)}_{k\in S}$ is any optimal solution to *IP*(*λ^ℓ^*). The step length is computed according to formula

$$\mu_{\ell }=\frac{\theta_{\ell }\left(UB-z\left(\lambda^{k}\right)\right)}{{\left\|\Sigma_{k\in S}\;y^{k}\left(\ell \right)-P\right\|}^{2}},$$

where *UB *is a previously computed upper bound on z* and *θ *is a step size parameter assuming values in {*x *∈ ℝ | 0 <*x *≤ 2}. In the experiments it turns out, that initializing the vector of Lagrangian multipliers *λ*^0 ^to the length *P *of the corresponding intervals in P increases the convergence rate dramatically. We also experienced a fast convergence to near-optimal Lagrangian multipliers when following the classical Held-Karp method to choose the step size scalar *θ*: We start with *θ*_0 _= 2 and half *θ*_ℓ _whenever the best Lagrangian bound *v*(*IP*(*λ*)) found so far has not increased in a certain number of iterations. As soon as the step size scalar falls below a specified threshold or the number of iterations exceeds a certain limit (which is adaptive with respect to the depth of the branch-&-bound node), we branch on a variable $y_{p}^{k},\;k\in S,\;p\in P$, such that${\overset{ª}{y}}_{p}^{k}-\left\lfloor {\overset{ª}{y}}_{p}^{k}\right\rfloor$ is close to 0.5, where ${\overset{ª}{y}}_{p}^{k}$ is the average value of variable $y_{p}^{k}$ in the last *h *= 10 Lagrangian solutions. Since we aim to find *all *optimal colorings, we also branch on variables that are integral. Incorporating the Lagrangian approach as a lower bounding scheme into a branch-&-bound frame work gives an alternative algorithm that does not depend on commercial software packages.

### Experimental Setting

The entire sHDX experiment was automated with a LEAP robot (HTS PAL, Leap Technologies, Carrboro, NC).

Automation of the experiment reduces human error and reduces deuterium for hydrogen back-exchange. All time points where interlaced and performed in triplicate to ensure experimental reproducibility. After digestion, the protein digest was injected from a 10 *μ*L loop to either a 1 mm × 50 mm C5 column (Phenomenex) or a Pro-Zap Pro-sphere HP C18 HR 1.5u 10 mm × 2.1 mm (All-tech). A rapid gradient 2% B to 95% B in 1.5 min(A: acetonitrile/H_2_O/formic acid 5/94.5/0.5, B: ace-tonitrile/H_2_O/formic acid 95/4.5/0.5) was used to elute peptides. The eluent was post-column split and infused by microelectrospray ionization into a custom built 14.5 T LTQ FT-ICR mass spectrometer. The extraction of the peptic fragments and their deuterium uptakes from these data was done by an in-house analysis package [25]. Then we compute the cumulative exchange rates from the deuterium uptakes with either the MEM-method [15] or a new approach based on integer linear programming [14].

A current limitation for implementation of this software is back exchange of deuterium-to-hydrogen during the separation of the samples. It has been reported that different peptides have a different percentage of back exchange due to the sequence of amino acids [26,27]. Furthermore, the peptide sequence overlap will limit the ability to map single amino acid rate kinetics. Thus, reduction of backexchange has been investigated [28,29], along with multiple acid proteases to increase sequence coverage [30]. The sHDX experiment is continually being improved, but in its current state the sHDX experiment does not take away from the integrity of the algorithm to discern single amino acid exchange kinetics.

## Authors' contributions

HMZ, JT, and MRE performed the H/D exchange experiments and analyzed the data to yield the rate constant distributions from which the subsequent residue assignments were made. EA, SC, CE, and AK developed the mathematical model, performed the computational experiments and drafted the manuscript. AMB initiated and identified the mathematical approach. AGM participated in the design and coordination of the study and in preparation of the manuscript. All authors read and approved the final manuscript.
